# Protocol for multiplexed RNAscope-based imaging of mRNAs in whole-mount adult *Drosophila* brains

**DOI:** 10.1016/j.xpro.2025.103615

**Published:** 2025-01-31

**Authors:** Meilin Wu, Vanessa Lambatan, Peng Guo, William J. Joiner

**Affiliations:** 1 Department of Pharmacology, University of California, San Diego, La Jolla, CA 92093, USA; 2 Center for Circadian Biology, University of California, San Diego, La Jolla, CA 92093, USA; 3 Department of Cellular and Molecular Medicine, University of California, San Diego, La Jolla, CA 92093, USA; 4 Nikon Imaging Center, University of California, San Diego, La Jolla, CA 92093, USA

**Keywords:** single cell, microscopy, gene expression

## Abstract

Visualizing the expression of mRNAs using traditional *in situ* hybridization is often hampered by obstacles including weak signal, high background, and poor probe specificity. Here, we present a protocol utilizing RNAscope (ACD) to overcome these obstacles and detect multiple types of mRNAs simultaneously in whole-mount adult *Drosophila* brains. We further describe how mRNAs can be reliably quantified in any cells that can be targeted by common binary expression systems such as Gal4/UAS and labeled by immunohistochemistry.

For complete details on the use and execution of this protocol, please refer to De et al.^1^

## Highlights

• Protocol for multiplexed RNAscope and IHC labeling in whole-mount *Drosophila* brains
• Steps for tissue preparation, transcript labeling, and IHC
• Instructions for quantitative analysis of transcript levels in labeled cells

## Abstract (editor highlights)

Publisher’s note: Undertaking any experimental protocol requires adherence to local institutional guidelines for laboratory safety and ethics.

## Graphical abstract

**Figure:**
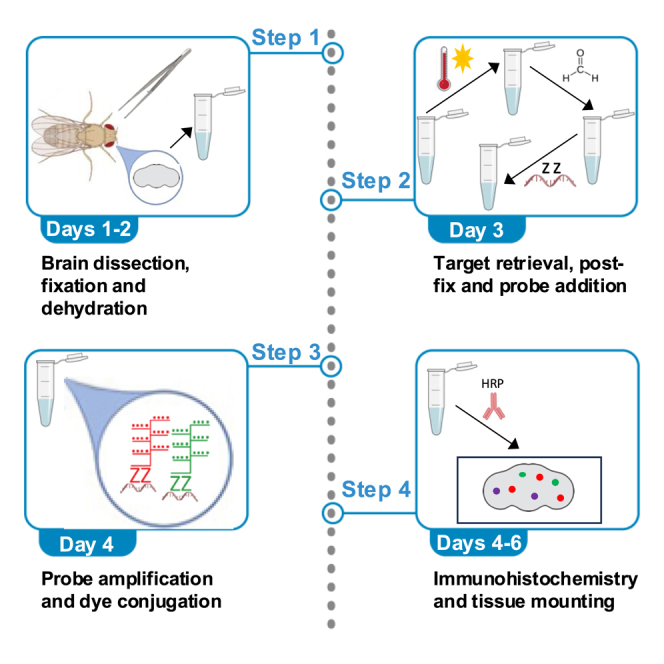

## Before you begin

The protocol below is based on the notion that RNAscope is sufficiently sensitive to detect individual molecules of mRNA in fixed cells (Xie et al.^2^). Our protocol has been modified from the technical notes provided with ACDBio’s Multiplex Fluorescent Detection Kit v2 and from work done in zebrafish embryos (Gross-Thebing et al.^3^). It has been adapted for whole mount staining of adult *Drosophila* brains and combined with immunostaining of genetically labeled cell populations using Gal4/UAS expression of a hemagglutinin-tagged GFP marker (De et al.^1^).

### Order probes

Prior to beginning, it is important to determine which RNAscope probes will be used and to prepare the appropriate reagents. When ordering a probe, it must be assigned to one of three channels (C1, C2, C3), each of which will ultimately be matched with a particular Opal dye. Therefore, when performing multiplexed imaging, care must be given to use probes with separate channels. If IHC labeling of GFP will also be used, then determine which Opal dyes will be used for each mRNA probe and secondary antibody, and check that the excitation/emission spectra are compatible.

### Tissue preparation and collection

Adult *Drosophila* of the desired genotypes should be reared according to desired experimental design. If comparing multiple genotypes, ensure that animals matched by age and sex. Brains can be dissected as previously described (De et al.^1^), fixed in 4% formaldehyde/PBS, and stored for up to 6 months at −20°C in 100% methanol after the dehydration step (Day 2).

### Prepare reagents

The following reagents can be prepared ahead of time unless indicated otherwise:

1. For brain dissections and dehydration/rehydration.
  a. 4% formaldehyde in 1X PBS (prepare fresh daily).
  b. 1X PBST (1X PBS + 0.3% Triton X-100; can be stored at 21°C–23°C).
  c. 25%, 50%, and 75% methanol in 1X PBST (can be stored at 21°C–23°C).
  d. 1X PBST + 1% BSA (store at 4°C).
2. Day 3 reagents.
  a. 1X Target Retrieval (dilute 10X Target Retrieval from ACDbio Multiplex Fluorescent Detection. Kit v2 in dH_2_O; prepare fresh daily and prewarm to 100°C immediately prior to use).
  b. 4% formaldehyde in 1X PBS (prepare fresh daily).
  c. RNAscope probes: prewarm for 10 min at 40°C; ensure that solutions are completely solubilized (crystals can form during prolonged storage at 4°C).
3. Day 4 reagents.
  a. Opal dyes: dilute appropriate Opal dyes in TSA buffer (ACDBio Multiplex Fluorescent Detection Kit v2) typical dilutions range from 1:750 to 1:2000 and can be stored in the dark at 4°C for up to 1 month).
  b. IHC blocking buffer: 10% normal goat serum + 1% BSA + 0.3% Triton X-100 in dH_2_O (can be stored at 4°C).
  c. Primary antibody for IHC should be diluted in IHC blocking buffer and can be stored at 4°C.
4. Additional IHC reagents.
  a. HRP-conjugated secondary antibodies should be diluted in IHC blocking buffer and prepared fresh daily.
  b. Opal dyes: dilute and store as described above.

**CRITICAL:** It is important to ensure that the combination of probes and Opal Dyes are compatible. For example, three probes can be multiplexed to target three different mRNA transcripts as long as they are in different channels (i.e., channels 1, 2, or 3). For multiplexing 3 probes with additional IHC, we have found that combining the following 4 Opal dyes yields the best separation of wavelengths with minimal bleed-through upon imaging: Polaris Opal 480, Opal 540, Opal 620 and Opal 690.

## Key resources table

**Table:** 

REAGENT or RESOURCE	SOURCE	IDENTIFIER
**Antibodies**		

Rabbit anti-HA (1:600 dilution)	Rockland	RL600-401-384
Goat anti-rabbit-HRP (1:600 dilution)	VWR	95959–088
Mouse anti-AstA (1:100 dilution)	DSHB	5F10-c
Goat anti-mouse-HRP (1:600 dilution)	VWR	95059–094

**Chemicals, peptides, and recombinant proteins**		

Vectashield (or other mounting medium)	Vector Labs	H-1000
10X PBS	Fisher	BP665-1
Triton X-100	Fisher	BP151100
BSA	Gemini Bio-Products	700-100P
16% Formaldehyde	Thermo Fisher Scientific	PI28908
10% Goat serum	Invitrogen	50062Z
Methanol	Fisher	A4124

**Critical commercial assays**		

Multiplex Fluorescent Detection Kit v2	Advanced Cell Diagnostics	323110
RNAscope probe Dm-Sand-C2	Advanced Cell Diagnostics	573821-C2
RNAscope probe Dm-AstA-C3	Advanced Cell Diagnostics	573861-C3
Opal dye 540	Akoya Biosciences	FP1494001KT
Opal dye 690	Akoya Biosciences	FP1497001KT
Opal dye 620	Akoya Biosciences	FP1495001KT
Opal dye Polaris 480	Akoya Biosciences	FP1500001KT

**Experimental models: Organisms/strains**		

UAS-smGFP-HA,LexAop-smGFP-V5 (virgin female *Drosophila melanogaster*)	Bloomington Drosophila Stock Center	Stock number 64092
23E10-Gal4 (adult male *Drosophila melanogaster*)	Bloomington Drosophila Stock Center	Stock number 49032

**Software and algorithms**		

NIS Elements Analysis AR 5.41.01	Nikon	https://www.microscope.healthcare.nikon.com/products/software/nis-elements
Prism 8	GraphPad	https://www.graphpad.com

**Other**		

Adjustable speed rocker set at the lowest speed (approximately 1 rev/second)	N/A	N/A
Heat blocks set to 40°C and 100°C	N/A	N/A
Nikon Ti2-E microscope with CREST X-Light V2 spinning disc confocal scan-head	Nikon	https://www.microscope.healthcare.nikon.com

## Materials and equipment

***Note:*** All reagents described in the protocol are included in the Multiplex Fluorescent Detection Kit v2 except for those listed below.

**Table:** PBST

Reagent	Final concentration	Amount
10X PBS	1x	5 mL
10% Triton X-100	0.3%	1.5 mL
ddH_2_O	N/A	43.5 mL
**Total**	**N/A**	**50 mL**

Can be prepared in advance and stored at 21°C–23°C for several months.

**Table:** PBST + 1% BSA

Reagent	Final concentration	Amount
10X PBS	1x	5 mL
10% Triton X-100	0.3%	1.5 mL
10% BSA in dH_2_O	1%	5 mL
ddH_2_O	N/A	38.5 mL
**Total**	**N/A**	**50 mL**

Sterile filter for storage at 4°C for several months.

**Table:** IHC Blocking solution

Reagent	Final concentration	Amount
10% Triton X-100	0.3%	0.3 mL
10% BSA in dH_2_O	1%	1 mL
10% Normal Goat Serum	8.7%	8.7 mL
**Total**	**N/A**	**10 mL**

Can be prepared in advance and stored at 4°C for several months.

### 4% formaldehyde/PBS

1 volume of 16% formaldehyde in 3 volumes of 1X PBS. Prepare fresh from 16% formaldehyde stocks.

### Hazards

Formaldehyde can cause irritation of the skin, eyes, nose, and throat. Thus, it should be handled with care. Solutions should be prepared in a chemical hood with proper PPE to reduce exposure, and they should be properly disposed of.

### Methanol/PBST for dehydration and rehydration

prepare 3 methanol solutions in PBST: 25% methanol, 50% methanol and 75% methanol. Methanol solutions can be prepared in advance and stored at 21°C–23°C for up to 2 months.

## Step-by-step method details

### Day 1: Tissue dissection and fixation

**Timing: approximately 1–2 h**

This section briefly describes tissue collection and fixation.

1. Dissect adult *Drosophila* brains as previously described (De et al.^1^).
  a. Collect up to 6 brains in a 1.7 mL Eppendorf tube with 500–800 μL of 4% formaldehyde/PBS for fixation.
  b. Incubate in 4% formaldehyde/PBS 18 h at 21°C–23°C on a rocker set at the lowest speed.

### Day 2: Tissue dehydration

**Timing: approximately 1 h**

This section details tissue dehydration and optional long-term storage.

2. With a 1 mL pipette tip, carefully remove formaldehyde/PBS solution.
  a. Wash for 10 min at 21°C–23°C on a slow rocker in 650 μL PBST.
3. Dehydrate brains stepwise in methanol/PBST solutions.
  a. Replace wash solution with 500–600 μL of 25% methanol/PBST. Incubate for 10 min at 21°C–23°C on a slow rocker.
  b. Replace 25% methanol solution with 500–600 μL of 50% methanol/PBST. Incubate for 10 min at 21°C–23°C on a slow rocker.
  c. Replace 50% methanol solution with 500–600 μL of 75% methanol/PBST. Incubate for 10 min at 21°C–23°C on a slow rocker.
  d. Replace 75% methanol solution with 500–600 μL of 100% methanol. Incubate for 10 min at 21°C–23°C on a slow rocker.
  e. Remove 100% methanol and replace with 500 μL of fresh 100% methanol.
  f. Store dehydrated brains at −20°C.

**Pause point:** [Dehydrated brains can be stored in 100% methanol at −20°C for up to 6 months].

### Day 3: RNAscope multiplex probe labeling

**Timing: 2–3 h**

This section describes processing of the brains for probe hybridization.

***Note:*** prepare fresh 1X Target Retrieval and 4% formaldehyde post fix solutions.

4. Rehydrate brains stepwise in methanol/PBST solutions.
  a. Recover the brains from frozen storage and allow to return to 21°C–23°C.
  b. Replace 100% methanol with fresh (21°C–23°C) 100% methanol.
  c. Replace 100% methanol with 500–600 μL of 75% methanol/PBST. Incubate for 10 min at 21°C–23°C on a slow rocker.
  d. Replace 75% methanol with 500–600 μL of 50% methanol/PBST. Incubate for 10 min at 21°C–23°C on a slow rocker.
  e. Replace 50% methanol with 500–600 μL of 25% methanol/PBST. Incubate for 10 min at 21°C–23°C on a slow rocker.
  f. Replace 25% methanol and wash with 650 μL of PBST + 1% BSA for 10 min at 21°C–23°C on a slow rocker.
5. Target Retrieval.
  a. Pre-heat 1X Target Retrieval solution to 100°C in a heat block.
  b. Remove PBST + 1% BSA wash solution. Replace with ∼500 μl of pre-heated 1X Target Retrieval solution and incubate for 5 min 30 s in a 100°C heat block.
  c. Remove 1X Target Retrieval solution and rinse for 1 min at 21°C–23°C in 650 μL PBST + 1% BSA.
  d. Remove PBST + 1% BSA wash solution and rinse for 1 min at 21°C–23°C in 650 μL 100% methanol.
    ***Note:*** Solution may become cloudy at this point.
  e. Remove 100% methanol and wash for 10 min at 21°C–23°C in 650 μL PBST + 1% BSA on a slow rocker.
6. Post-Fix.
  a. Remove wash solution and replace with 600–800 μL of fresh 4% formaldehyde/PBS. Incubate for 25 min at 21°C–23°C on a slow rocker.
    ***Note:*** This is a good time to prepare probes.
  b. Wash with 650 μL PBST + 1% BSA for 10 min at 21°C–23°C on a slow rocker.
7. Protease Plus.
  a. Remove as much of the wash solution as possible. Replace with 2–3 drops (approximately 50–100 μL) of Protease Plus. Incubate for 10 min in a 40°C heat block.
  b. Wash for 10 min at 21°C–23°C in 650 μL PBST + 1% BSA.
8. Add premixed probes.
  a. Remove as much of wash solution as possible. Replace with 2–3 drops (approximately 50–100 μL) of pre-warmed probe diluent.
  b. Remove as much probe diluent as possible. Replace with ∼50–100 μL of pre-mixed probes.
  c. Incubate 18 h in a 40°C in heat block.

***Note:*** Pre-warm probe diluent and probes for 10 min at 40°C. Check that all particulate precipitates are re-solubilized before mixing probes. If using a C1 probe, use ∼50–100 μL. If using additional C2 and/or C3 probes, dilute them 1:50 in the C1 probe. If using only C2 or C3 probes, dilute them 1:50 in pre-warmed probe diluent. For multiple samples using the same probe combinations, make enough pre-mixed probe for ∼50–100 μL per sample as a master mix and then distribute equally to each sample. For example, for 3 samples using the same probe combination, prepare ∼150–300 μL of pre-mixed probe and distribute among all three samples.

**CRITICAL:** It is very important that all the brains are submerged and freely floating (not clumped or sticking to the side of the tube at all steps. Brains will become very translucent during sample processing, so it is important to visually inspect the samples at each wash to ensure no tissue is lost. Best results are achieved by inverting the tube at each wash and visually confirming that all brains sink to the bottom prior to removing wash or reagent solutions.

### Day 4: Signal amplification and Opal dye conjugation

**Timing: 4.5–10 h**

This section closely follows the manufacturer’s instructions for Multiplexed RNAscope labeling. Amplification steps expand the binding sites on the probe for dye conjugation. Each probe channel is paired with a dye of the user’s choice in sequential steps. All wash steps in this portion of the protocol are in 1X RNAscope Wash Buffer (RWB; provided in kit at 50X concentration; diluted to 1X in dH_2_O). All steps after addition of the first Opal dye are done in the dark to prevent bleaching.

***Note:*** Opal dyes should be diluted in TSA buffer at concentrations ranging from 1:750 (max) to 1:2000.

9. Amplification.
  a. Carefully remove probe solution after 18 h incubation in a 40°C heat block.
  b. Wash 2 × 5 min at 21°C–23°C in 650 μl 1X RWB.
  c. Remove as much of wash solution as possible and replace with 2–3 drops (approximately 50–100 μL) of AMP 1 solution. Incubate for 30 min in a 40°C heat block.
  d. Wash 2 × 5 min at 21°C–23°C in 650 μL 1X RWB.
  e. Remove as much of wash solution as possible and replace with 2–3 drops (approximately 50–100 μL) of AMP 2 solution. Incubate for 30 min in a 40°C heat block.
  f. Wash 2 × 5 min at 21°C–23°C in 650 μl 1X RWB.
  g. Remove as much of wash solution as possible and replace with 2–3 drops (approximately 50–100 μL) of AMP 3 solution. Incubate for 15 min in a 40°C heat block.
  h. Wash 2 × 5 min at 21°C–23°C in 650 μL 1X RWB.
10. Opal Dye conjugation for channel 1 probe (if no C1 probe was used, skip to C2 or C3 dye conjugation).
  a. Remove as much of the wash solution as possible. Replace with 3 drops (approximately 100 μL) of HRP C1 solution. Incubate for 15 min in a 40°C heat block.
  b. Wash 2 × 5 min at 21°C–23°C in 650 μL 1X RWB.
  c. Replace with 200–400 μL of diluted Opal Dye #1. Incubate for 30 min in a 40°C heat block. Be sure to keep track of which dye is used for each channel.
  d. Wash 2 × 5 min at 21°C–23°C in 650 μL 1X RWB.
  e. Replace with 2–3 drops (approximately 50–100 μL) of HRP blocker solution. Incubate for 15 min in a 40°C heat block.
  f. Wash 2 × 5 min at 21°C–23°C in 650 μL 1X RWB.

***Optional:*** Repeat Opal Dye conjugation step for each probe used. For example, in a three probe multiplexed experiment, HRP-C1/Opal Dye #1 is followed by the same steps for HRP-C2/Opal Dye #2 and so on. HRP/Opal dye conjugation can be skipped for channels where no probe was used.

***Note****:* HRP-Opal dye conjugation for each channel takes about 1.5–2 h.

11. At this step, RNAscope labeling is complete and, if no IHC labeling is required, the brains can be mounted on glass slides in Vectashield or other mounting medium, then sealed with a glass coverslip and clear nail polish (as previously described; De et al.^1^).

***Note:*** If Multiplexed IHC labeling is desired, continue to next steps before mounting brains.

### Days 4–6: Antibody labeling for multiplexed IHC

**Timing: 1.5–2 h on day 4, 1 h on day 5, 2–3 h on day 6**

This section follows standard IHC antibody labeling protocols. However, according to the RNAscope kit manufacturer, the above protocol is less compatible with standard Alexa-Fluor pre-conjugated secondary antibodies. Instead, following primary antibody binding, use HRP-conjugated secondary antibodies allowing for subsequent Opal Dye conjugation. This portion of the protocol uses both 1X PBST and 1X RNAscope Wash Buffer (RWB) for washing steps.

12. Block tissue samples for IHC labeling.
  a. Wash 3 × 10 min at 21°C–23°C in 1X PBST.
  b. Block for 1 h at 21°C–23°C in Blocking solution [10% NGS, 1% BSA, 0.3% Triton X-100]. Alternatively, samples can be blocked 18 h at 4°C.
13. Primary antibody incubation.
  a. Dilute primary antibody in Blocking Solution. For R-anti-HA (1:600), for M-anti-AstA (1:100).
    ***Note:*** Primary antibodies can often be re-used several times.
  b. Replace blocking solution with 600–800 μL of diluted primary antibody.
  c. Incubate for 24–48 h at 4°C on a slow rocker.
    ***Note:*** Depending on primary antibody, incubation can be shortened to 2–4 h at 21°C–23°C , but we have observed optimal results with longer incubations. In addition, labeling can vary from antibody to antibody. We find that high affinity antibodies against many commercial epitopes (e.g. HA, V5, Flag) work well under the conditions described above, especially for strong expression of transgenes using the Gal4/UAS system. However, some antibodies may not work as well under the harsh conditions of this protocol.
14. Secondary antibody incubation and Opal dye conjugation.
  a. Dilute HRP-conjugated secondary antibody (1:600) of the appropriate species [e.g., goat anti-rabbit-HRP if using a rabbit primary antibody] in Blocking solution.
  b. Carefully remove primary antibody with a pipette and store at 4°C if planning to re-use.
  c. Wash 4 × 15 min at 21°C–23°C in 1X PBST.
  d. Add diluted secondary antibody and incubate 18 h at 4°C on a slow rocker.
15. Opal Dye conjugation.
  a. Remove secondary antibody solution and wash 1X in PBST.
  b. Wash 2 × 5 min in 650 μL of 1X RWB.
  c. Replace with 200–400 μL of diluted Opal dye. Incubate for 30 min in a 40°C heat block.
    ***Note:*** Be sure to use a dye that is not the same as dyes used to label transcripts.
  d. Wash 2 × 5 min in 650 μL of 1X RWB.
  e. Remove as much of the wash buffer as possible. Replace with 2–3 drops (approximately 50–100 μL) of HRP-blocker. Incubate for 15 min in a 40°C in heat block.
  f. Wash 2 × 5 min in 650 μL of 1X RWB.
16. Mount brains.
  a. Remove brains from wash buffer and mount on a glass slide in Vectashield or other mounting medium. Cover with a glass coverslip and seal with clear nail polish.

***Note:*** Mounted samples can be stored short-term at 4°C or long-term at −20°C. Opal Dye fluorescence seems to decay unevenly across various wavelengths, so for quantitative measurements, it is best to collect images within less than 1 week of staining. If multiple antibody labeling is desired, antibodies must be from different host species. The entire process of Days 4–6 must be repeated for each new antibody using a different Opal dye. While the protocol described above does not use a nuclear stain, DAPI is included in the Multiplex Fluorescent Detection kit v2 from ACD and can be used according to the manufacturer’s instructions. In this case, avoid using Opal 480 labeling as this will interfere with the DAPI signal.

### Image acquisition

**Timing: several hours, depending on number of wavelengths, image resolution, and sample number**

This section provides steps and parameters for confocal imaging of whole mount adult *Drosophila* brains following multiplexed RNAscope and IHC.

***Note:*** The details below are applicable to a CREST X-Light V2 spinning disc confocal scan-head mounted on Nikon Ti2-E microscope using a 60X 1.2NA water immersion objective and Hamamatsu Orca Fusion sCMOS camera, but similar results should be achievable using other imaging systems. For quantitative puncta analysis, images are acquired with 0.25 μm Z-steps at 0.11 μm/pixel resolution. Acquisition at 16 bit depth allows for a maximum intensity of 65,000 shades in each channel, ensuring that signal saturation is unlikely. Be aware that images acquired at this resolution and with four wavelengths can result in very large image files (from 3–7 GB per stack).

17. Adjust the laser intensity and exposure time for each wavelength. Use the following lasers for each Opal Dye: Polaris 480: 446 nm; Opal 540: 526 nm; Opal 620: 540 nm; Opal 690: 640 nm.

***Note:*** Ensure that the signal is not saturated and that it allows for some dynamic range. If the signal in one channel is extremely bright, faint bleed-through can sometimes be observed in other channels, especially between Opal 620 and Opal 690. Be sure to check that all signal is coming from your correct channel. Single dye experiments for each probe can be run and then imaged with all the target wavelengths for future multiplexing as a negative control for bleed-through. If comparing separate samples, make sure to use the same laser and exposure settings for all samples.

18. Set the upper and lower boundaries of the desired Z-stack.
19. Image each wavelength sequentially rather than in parallel to reduce bleed-through artifacts.

### Image analysis and quantification

**Timing: several hours to days**

This section provides details for image processing using Nikon’s NIS Elements software. However, other imaging analysis software such as ImageJ or Imaris should be able to perform similar analysis.

**Figure 1:**
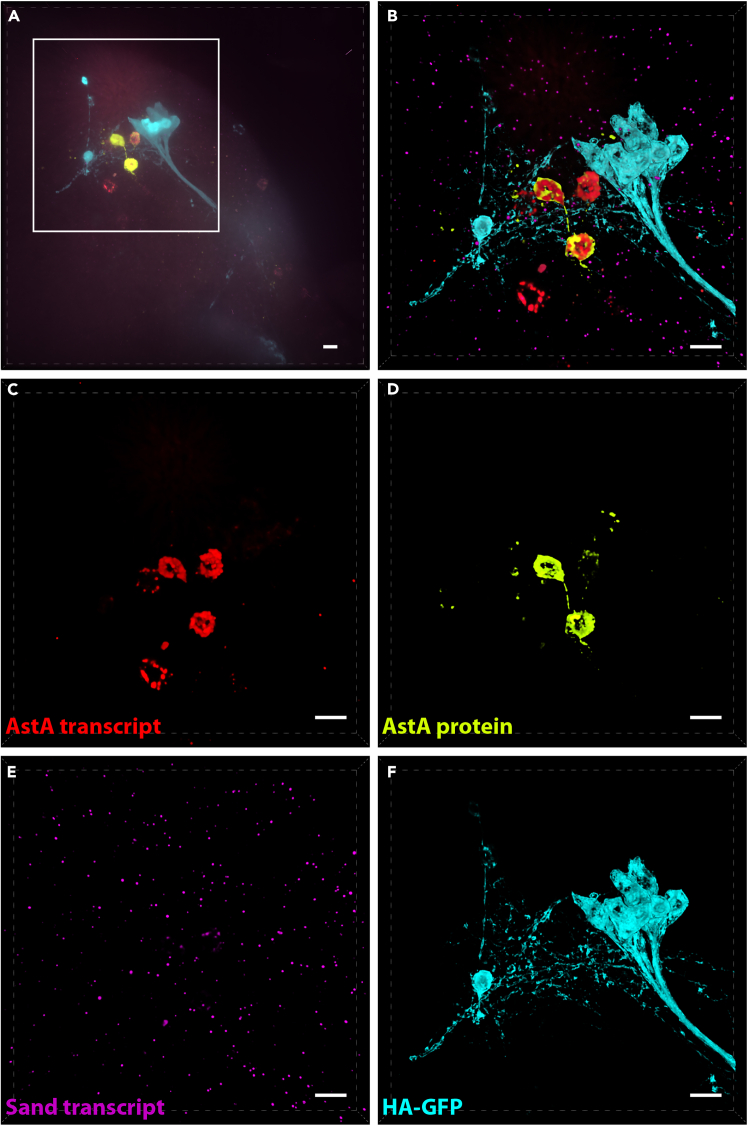
Volume projections of whole-mount adult 23E10>HA-GFP *Drosophila* brains with multiplexed labeling (A) Raw 60X volume projection prior to cropping and deconvolution. *AstA* transcript (Red), AstA protein (yellow), *Sand* transcript (purple) and HA-GFP (blue). (B) Volume projection of cropped and 3D deconvolved stack with merged channels. (C–F) Volume projections of individual channels in (B). All scale bars are 10 μm.

20. Image Processing.
  a. Optional: Perform 3D deconvolution to remove out of focus signal (compare Figures 1A and 1B–1F).

***Note:*** Numerous 3D deconvolution software packages are available, which can generally be categorized into two main approaches. The first is a subtractive method, which offers a fast yet qualitative approach to denoising by subtracting presumed out-of-focus photos through theoretical estimation. The second approach is quantitative and involves redistributing photons from out-of-focus planes to their correct focal planes. This is achieved using either a theoretical or experimentally measured point spread function (PSF). In this study, we employed a theoretical PSF calculated based on the optical system’s parameters, including wavelength, numerical aperture, and refractive index, to reassign photons. This method produced sharper images with reduced background noise. However, it is crucial to carefully manage the number of processing iterations to avoid introducing artifacts such as ringing or noise enhancement. For this work, Nikon Elements software was utilized. However, other commercial software such as Huygens or Imaris ClearView, as well as open-source alternatives such as Fiji/ImageJ can perform similar functions.

**Figure 2:**
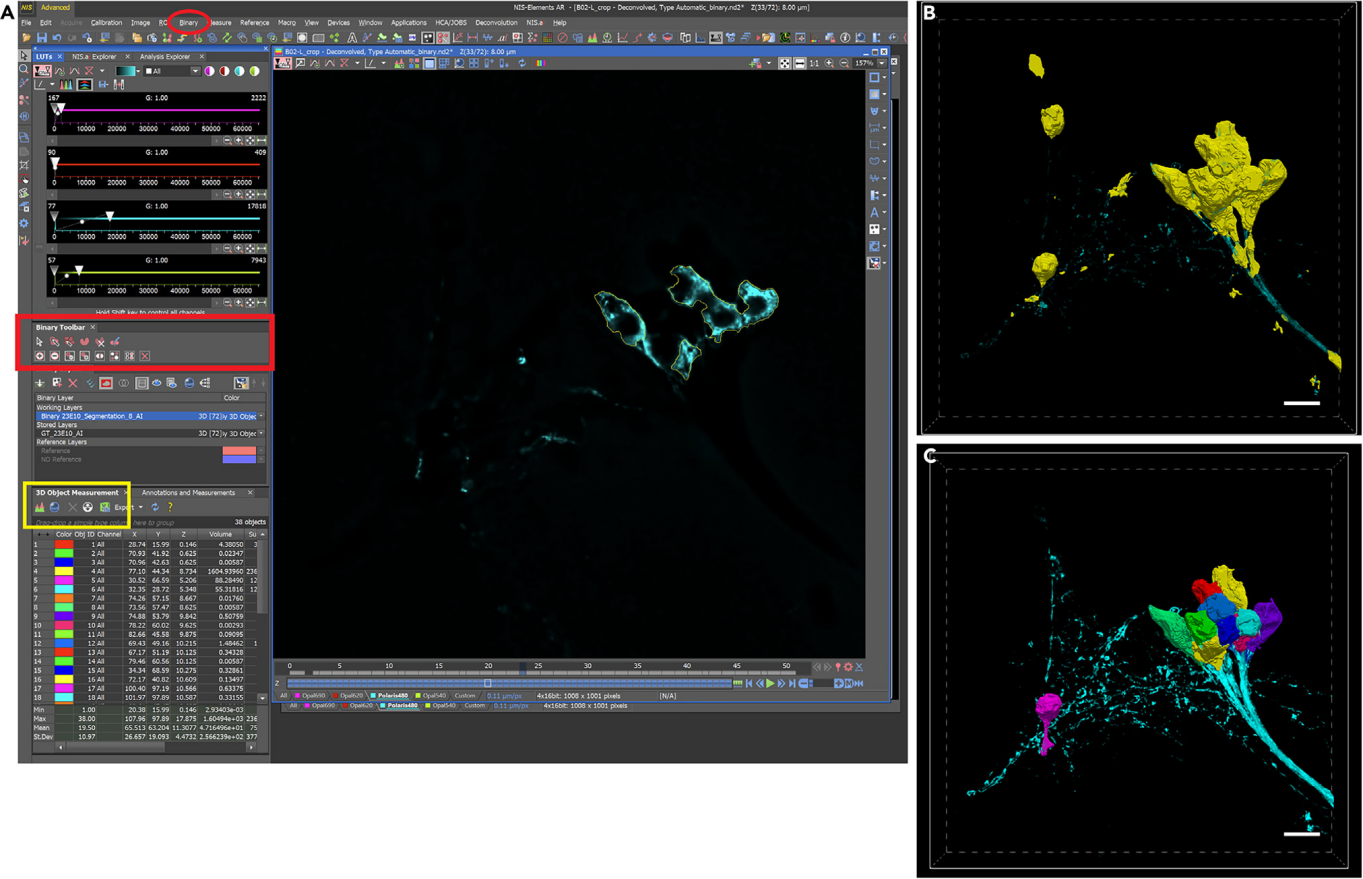
Labeling cell volumes with NIS-Elements AR using binary masks (A) Single z-slice view with HA-GFP signal highlighted (yellow outline) in a binary layer. Editing of the binary layer can be done using the Binary Toolbar (left, red box) or the Binary Editor pull-down menu (Top toolbar, red circle). 3D objects are generated using the “connect 3D objects” function in the 3D Object Measurements toolbar (bottom left, yellow box). (B) Volume projection view of fully labeled binary mask (yellow) of HA-GFP signal. (C) Completed manually edited binary layer with each cell volume delineated as a separate 3D Object. All scale bars are 10 μm.

**Figure 3:**
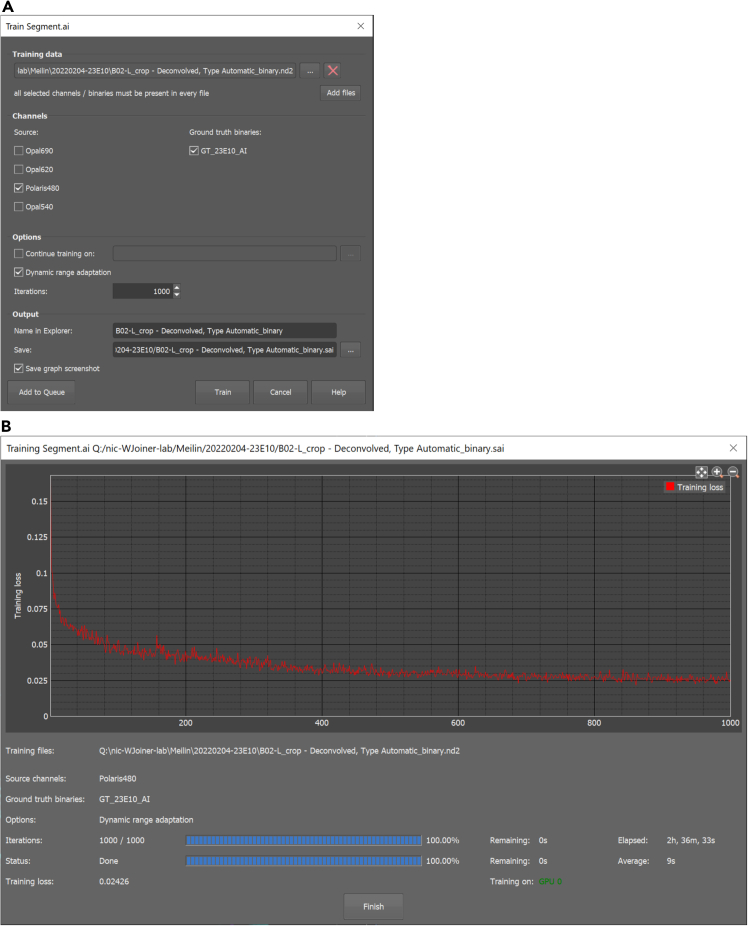
Training NIS.ai to automatically segment objects (A) NIS.ai Segment ai training interface. Using a fully edited ground truth binary, NIS.ai can be trained to automatically label a binary mask based on several parameters, including the source channel. A single ground truth is sufficient data for training, but multiple ground truth binaries can be input as training data. (B) An example plot of training loss over 1000 iterations of training.

**Figure 4:**
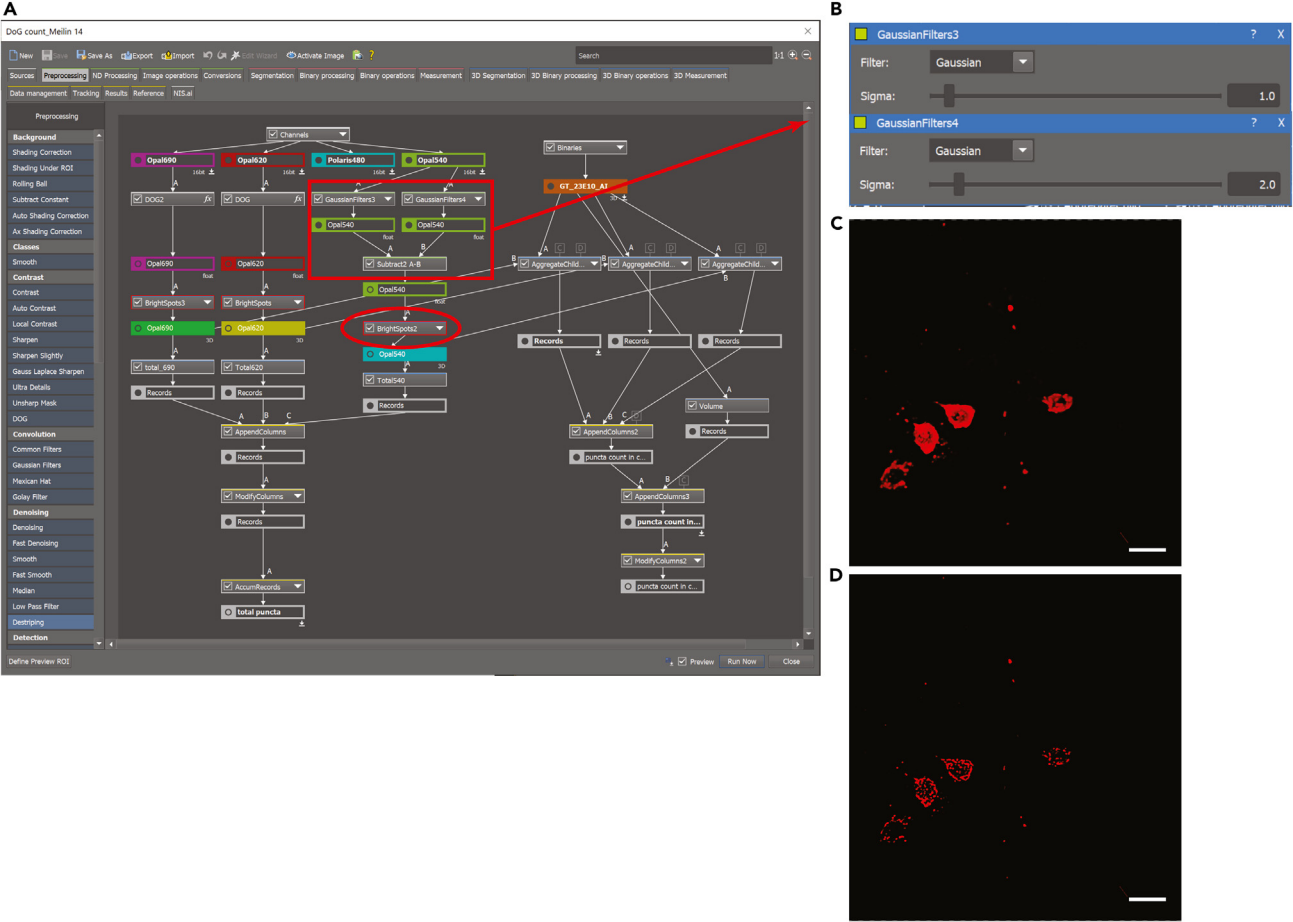
DOG filter and Bright Spot thresholding (A) Overview for NIS elements image processing workflow. Three channels (Opal 690, Opal 620, and Opal 540) were processed in parallel through a Difference of Gaussian (DOG) filter and Bright Spot detection/counting steps. (B) DOG parameters. (C) Volume projection image RNAscope labeled *AstA* transcript (Opal 620) after deconvolution. (D) Image after application of DOG filter. All scale bars are 10 μm.

**Figure 5:**
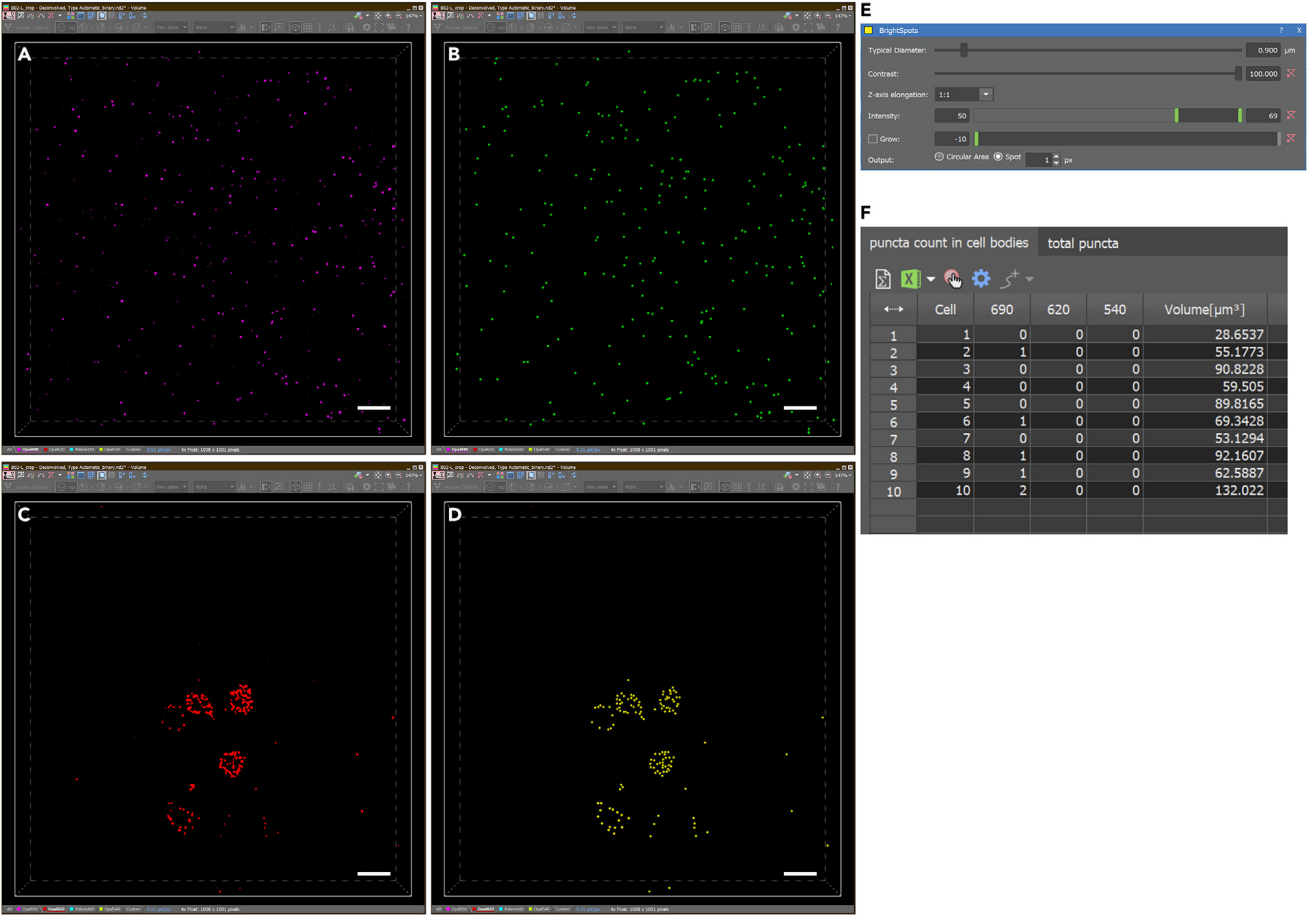
Bright Spot detection and counting after DOG filter (A) DOG filtered volume projection of Opal 690 signal (*Sand*, purple). (B) Bright Spot detection of DOG filtered 690 signal. Green dots represent each bright spot (shown at 5 pixels/spot for visibility). (C) DOG filtered volume projection of Opal 620 signal (*AstA*, red). (D) Bright Spot detection of DOG filtered 620 signal. Yellow dots represent each bright spot (shown at 5 pixels/spot for visibility). (E) Bright Spot detection parameters. (F) Quantification of the number of bright spots in each channel in each cell body defined by 23E10>HA-GFP (not shown; see Figure 2). All scale bars are 10 μm.

**Figure 6:**
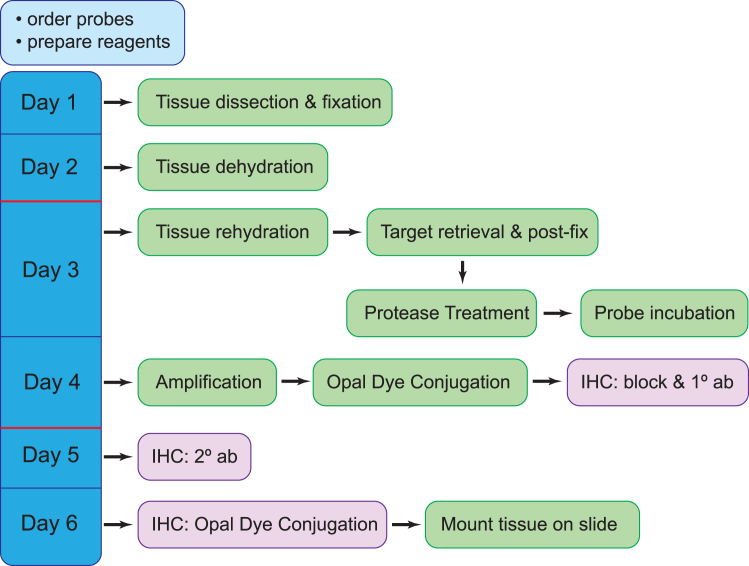
Flowchart summarizing RNAscope protocol Preparatory steps are shown at top left (light blue). Each step in performing RNAscope (green) is shown to the right of the day on which it is performed (dark blue). Optional steps for performing IHC are shown in purple. Red lines indicate recommended pause points.

21. Binary Masking and Cell Segmentation – Analysis using NIS-AS software.
  ***Note:*** The details below are optional but useful for speeding up the process of defining antibody-labeled target cells for quantification of transcript within the cells. Using the NIS.ai to streamline binary mask labeling of target cells can save many hours of image processing when quantifying transcripts in large populations. If only small numbers of cells need to be analyzed, binary masks can be generated by simple thresholding of the antibody labeled signal.
  a. Generate a “ground truth” binary by manually delineating areas labeled as a “cell” by IHC to facilitate training of an AI for subsequent automated binary labeling of cells.
    ***Note:*** This can be done using the NIS elements tools in the Binary Toolbar as well as in the Binary Editor mode. Auto-selection of thresholded signal can be a useful starting point. However, it is usually necessary to follow up manually using the binary editing tools to remove false positive selections and to highlight areas with signal that automated thresholding missed. (See Figure 2 for manually edited example ground-truth binary). Manual editing should result in accurate labeling of the IHC signal for each cell encompassed by a 3D binary object, which is generated by connecting 3D objects in the 3D object measurement toolbar (see Figure 2A, yellow box).
  b. After generating at least 1 ground truth binary, use NIS.ai to train “segment ai” for automated binary labeling. Several rounds of 1000x training sessions may be required to achieve acceptable results. When training-loss approaches 0.02, the AI should give acceptable results.
    ***Note:*** For multiple ground truths, where signals show wide variation in intensity or other variables, achieving a training-loss of 0.02 may not be possible (See Figure 3).
  c. Subsequent image files can be automatically processed for cell segmentation using trained segment AI programs, but they will likely still need to be manually edited using the binary toolbar for accuracy.
22. Puncta Quantification.
  a. Bright spots representing labeled transcripts in each channel are resolved after applying a Difference of Gaussian (DOG) filter (Figures 4A and 4B). Bright spots representing labeled transcripts in each channel are resolved after applying a Difference of Gaussian (DOG) filter. This process is a two-stage edge detection process. It performs two different Gaussian blurs using two blurring factors (standard deviations) on the image then uses the difference in the two blurred images as the final image to detect local maxima as bright spots (compare original deconvolved image in Figure 4C to DOG filtered image in Figure 4D. Bright spots in each wavelength are thresholded (Figures 5A–5D) using three parameters: 1. Typical diameter, 2. Contrast, and 3. Intensity (see Figure 5E). Spot size is set at the lowest setting (1 pixel/spot). Once thresholds are set, all images are quantified using the same setting to allow for comparison between samples.
    ***Note:*** To visually verify accuracy of spot detection, digital spot size may need to be increased to be visible. This can affect the number of spots counted inside each “cell” because spots that fall on the border are not included. Thus, it is important to return the digital spot size to 1 pixel prior to the final count so that the spot does not artificially overlap with the border, which would cause them to be unnecessarily excluded from the cell volume.
  b. Bright spots are then aggregated within each 3D binary object (corresponding to a cell) in order to be quantified as puncta (i.e., transcript) per cell for each probe (Figure 5F). Spots that fall on the border of a binary object are automatically excluded.
    ***Note:*** The DOG filter sharpens and brightens each spot such that images with reasonably low background will result in reliably quantifiable spots over a large range of signal intensity. However, it is important to visually verify several images for accuracy before setting final thresholds.
    A flowchart depicting all the steps in the protocol is shown in Figure 6.

## Expected outcomes

Successful multiplexed labeling and proper imaging of mRNAs combined with IHC of targeted cells should yield a multi-channel image file at high enough resolution to quantify individual transcripts (puncta) within cell bodies (3D binary object) (See Figure 5).

## Limitations

RNAscope labeling provides very strong signal with little to no noise. Furthermore, according to its developers, it is capable of detecting single mRNA molecules. This is a marked improvement over traditional ISH protocols where probe strength can be highly variable and subject to significant off-target labeling. Combining RNAscope with IHC labeling of genetically targeted cell populations allows for quantitative measurements of mRNA levels at a cellular level. However, the technique as described below does not account for mRNAs that are localized outside of the labeled cell body. For example, labeled mRNAs can be counted within a neuronal cell body, but not in distal processes. Furthermore, although individual mRNAs can be labeled and detected using this method, if transcript abundance is excessively high, it is theoretically possible that two transcripts might lie in close enough proximity that they would not be resolvable as two individual bright spots and would thus be counted as one punctum. This limitation would result in a possible undercounting of very abundant targets and thus, under such abundant expression scenarios, per-puncta quantification analysis might be challenging or impossible. Finally, in a few rare instances we have observed bleed-through from one channel to another when the signal is excessively bright. This is likely due to the very broad excitation/emission spectra of the Opal dyes. Special care should be taken to use the appropriate dyes and to ensure that bleed-through artifacts are avoided and/or excluded from puncta analysis.

## Troubleshooting

### Problem 1

Loss of tissue during labeling procedure. Brains will become very translucent as they are processed and are easily lost, especially during wash steps.

### Potential solution

• Visually inspect each sample and ensure each brain sinks to the bottom of the tube at each wash step. Brains can be counted as they sink to the bottom of the tube.
• Tissue integrity is aided greatly by the post-fix step (step 6) following target retrieval. Elimination of this step causes the tissue to be highly compromised and is thus essential to maintain brain structures throughout the remainder of the protocol. We have not observed any obvious alterations/deterioration of brain structures after following the steps described above.

### Problem 2

Uneven labeling across the brain.

### Potential solution

• Take care to see that all the brains are freely floating and not clumped together or stuck to the side of the tube at each step. This is particularly important during all the low volume incubations (probe, AMPs, HRP-C-1/2/3, and HRP blocker) to ensure even access to the reagents.
• We have not observed issues with probe penetration, but the target-retrieval step (step 5) is critical for subsequent antibody penetration in the IHC labeling. While we used the protease plus included with the kit for 10 min at 40°C, some probes might require additional testing of the different proteases and incubation times.

## Resource availability

### Lead contact

Further information and requests for resources and reagents should be directed to and will be fulfilled by the lead contact, William J. Joiner (wjoiner@health.ucsd.edu).

### Technical contact

Technical questions on executing this protocol should be directed to and will be answered by the technical contact, Meilin Wu (meilin@health.ucsd.edu).

### Materials availability

This study did not generate new unique reagents.

### Data and code availability

This study did not generate datasets or code.

## Acknowledgments

The authors thank Eric Griffis and the Nikon Imaging CORE for valuable advice and assistance in setting up image acquisition and analysis. This work was supported by NIH grants RO1 GM125080 and R21 NS123690 to W.J.J.

## Author contributions

M.W. and V.L. performed experiments. P.G. assisted in setting up image acquisition and analysis. M.W. wrote the manuscript with comments and editing by W.J.J., P.G., and V.L.

## Declaration of interests

The authors declare no competing interests.
